# The interrelationship between pregnancy, venous thromboembolism, and thyroid disease: a hypothesis-generating review

**DOI:** 10.1186/s13044-021-00102-4

**Published:** 2021-05-25

**Authors:** Stine Linding Andersen, Kasper Krogh Nielsen, Søren Risom Kristensen

**Affiliations:** 1 Department of Clinical Biochemistry, Aalborg University Hospital, Hobrovej 18-22, 9000 Aalborg, Denmark; 2 Department of Clinical Medicine, Aalborg University, Sdr. Skovvej 15, 9000 Aalborg, 9000 Aalborg, Denmark

**Keywords:** Pregnancy, Postpartum, Coagulation, Thrombosis, Thyroid

## Abstract

Pregnancy induces physiological changes that affect the risk of thrombosis and thyroid disease. In this hypothesis-generating review, the physiological changes in the coagulation system and in thyroid function during a normal pregnancy are described, and the incidence of venous thromboembolism (VTE) and thyroid disease in and after a pregnancy are compared and discussed. Furthermore, evidence regarding the association between thyroid disease and VTE in non-pregnant individuals is scrutinized. In conclusion, a normal pregnancy entails hormonal changes, which influence the onset of VTE and thyroid disease. Current evidence suggests an association between thyroid disease and VTE in non-pregnant individuals. This review proposes the hypothesis that maternal thyroid disease associates with VTE in pregnant women and call for future research studies on this subject. If an association exists in pregnant women specifically, such findings may have clinical implications regarding strategies for thyroid function testing and potential thromboprophylaxis in selected individuals.

## Introduction

A normal pregnancy comprises a series of physiological changes, which challenge the diagnosis of maternal diseases and affect the incidence of various diseases in and after a pregnancy. Changes in the coagulation system during a pregnancy include an increase in the levels of coagulation factors and fibrinolytic inhibitors, making pregnancy a hypercoagulable and hypofibrinolytic state. Thus, the risk of venous thromboembolism (VTE), i.e. deep venous thrombosis (DVT) and the complicating pulmonary embolism, increases gradually during pregnancy and peaks in the early postpartum period [1–3]. Furthermore, the anatomic changes during a pregnancy contribute to the risk as evidenced by the increased incidence of proximal left-sided VTE in pregnant women. Also, the function of the thyroid gland is considerably altered in pregnancy to ensure supply of thyroid hormone to the fetus. A predominant effect on thyroid function is mediated via the pregnancy hormone human chorionic gonadotropin (hCG), which stimulates the thyroid gland to an increased production of thyroid hormone [4]. Moreover, the changes in the immune system in and after a pregnancy may influence the onset of autoimmune thyroid disease [5–7]. Consequently, pregnancy entails numerous physiological changes capable of altering the coagulation system and the function of the thyroid gland. Adding to this, increasing evidence has indicated an association between abnormal thyroid function and abnormalities in the coagulation system in non-pregnant individuals [8–10].

We speculated on the possible interrelationship between pregnancy, VTE, and thyroid disease and performed a hypothesis-generating review on this subject. More specifically, we aimed to provide an overview of the physiological changes associated with a normal pregnancy in the coagulation system and in thyroid function and to describe the occurrence of VTE and thyroid disease in and after pregnancy. Furthermore, we aimed to describe the association between thyroid disease and VTE in non-pregnant individuals and to discuss possible mechanisms of their interaction in pregnancy. The review calls for future research studies that investigate the proposed hypothesis in pregnant women specifically. If an association exists, this could have clinical implications regarding strategies for thyroid function testing and thromboprophylaxis in selected individuals.

## The coagulation system in pregnancy

**Fig. 1:**
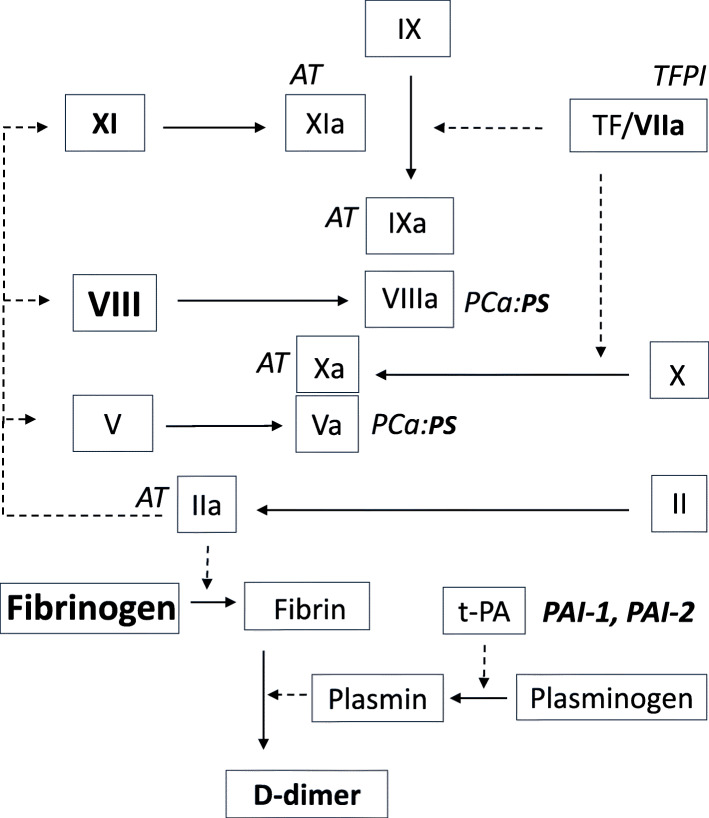
A simplified overview of the secondary hemostasis including the main alterations in pregnancy. Factors that considerably change in pregnancy are marked in bold (protein S (PS) decreases, others increase), and anticoagulants are marked in italic. See text for details. AT; antithrombin, PAI-1; plasminogen activator inhibitor-1, PAI-2: plasminogen activator inhibitor-2, PCa; activated protein C, TF: tissue factor, TFPI; tissue factor pathway inhibitor, t-PA; tissue-type plasminogen activator

Hemostasis consists of primary hemostasis, the coagulation system, and the fibrinolytic system. The primary hemostasis will establish a platelet plug. When the endothelial layer is damaged, von Willebrand factor (vWF) will bind to the subendothelial collagen structures enabling platelets to adhere, get activated and to form a platelet plug [11]. The secondary hemostasis results in the formation of a fibrin network to stabilize the plug (Fig. 1). It is activated via tissue factor (TF), which is present in the sub-endothelial layers [12]. TF forms a complex with coagulation factor VIIa, which in turn activates factors X and IX leading to the formation of thrombin (factor IIa) [12, 13]. Thrombin gives rise to a powerful positive feedback activation of factors V, VIII, and XI. Factors Va and VIIIa are cofactors for factors Xa and IXa, respectively, and the feedback mechanisms increase the enzyme activities more than 1000 times [13]. This gives rise to a large formation of thrombin that cleaves fibrinogen into fibrin successively forming a fibrin network. Factor XIIIa, activated by thrombin, catalyzes the formation of covalent bonds between the fibrin monomers that strengthen the plug [14, 15].

The activity of the coagulation system is balanced by endogenous anticoagulants (Fig. 1). Tissue factor pathway inhibitor (TFPI) in complex with factor Xa inactivates the TF/factor VIIa-complex [16]. Antithrombin (AT) inactivates thrombin and factors IXa, Xa, and XIa [17]. Finally, activated protein C (PCa) will, in complex with Protein S (PS), inactivate factors Va and VIIIa [18]. The fibrin network can be dissolved by the fibrinolytic system (Fig. 1). Mainly tissue-type plasminogen activator (t-PA), but also urokinase-type plasminogen activator (u-PA), can activate plasminogen to plasmin, which is an enzyme that catalyzes the degradation of fibrin to smaller parts, among others D-dimer [15]. t-PA and u-PA can be inhibited by plasminogen activator inhibitor-1 (PAI-1). During pregnancy, placenta produces another inhibitor, the so-called plasminogen activator inhibitor-2 (PAI-2) [15].

During pregnancy several changes lead to a procoagulant condition (Fig. 1), which is probably an evolutionary adaptation to protect against hemorrhage during the innate traumatic nature of giving birth. Especially the levels of vWF, fibrinogen, and factor VIII increase [19, 20], thus, they gradually increase from the first trimester to reach 2–3 times the non-pregnant level at delivery, and the levels normalize again a few weeks after birth of the child. The levels of factors VII and IX also increase, reaching levels of 30–50% above the non-pregnant state at delivery, whereas factors II, V, X, and XI are essentially unaltered [20]. In the anticoagulant system, the most marked change during pregnancy is a lowering of protein S which occurs early in pregnancy and the level of this anticoagulant is more than halved in second and third trimester [20–22]. In the fibrinolytic system, the inhibitors PAI-1 and PAI-2 gradually increase from the second trimester making pregnancy a hypofibrinolytic state [23, 24]. As a result of these changes, the routine laboratory analyses such as the activated partiel thromboplastin time (APTT) and prothrombin time (PT) may shorten slightly during pregnancy and postpartum, whereas D-dimer increases considerably, especially during the second and third trimester and immediately postpartum [20, 25].

## VTE in and after a pregnancy

**Fig. 2:**
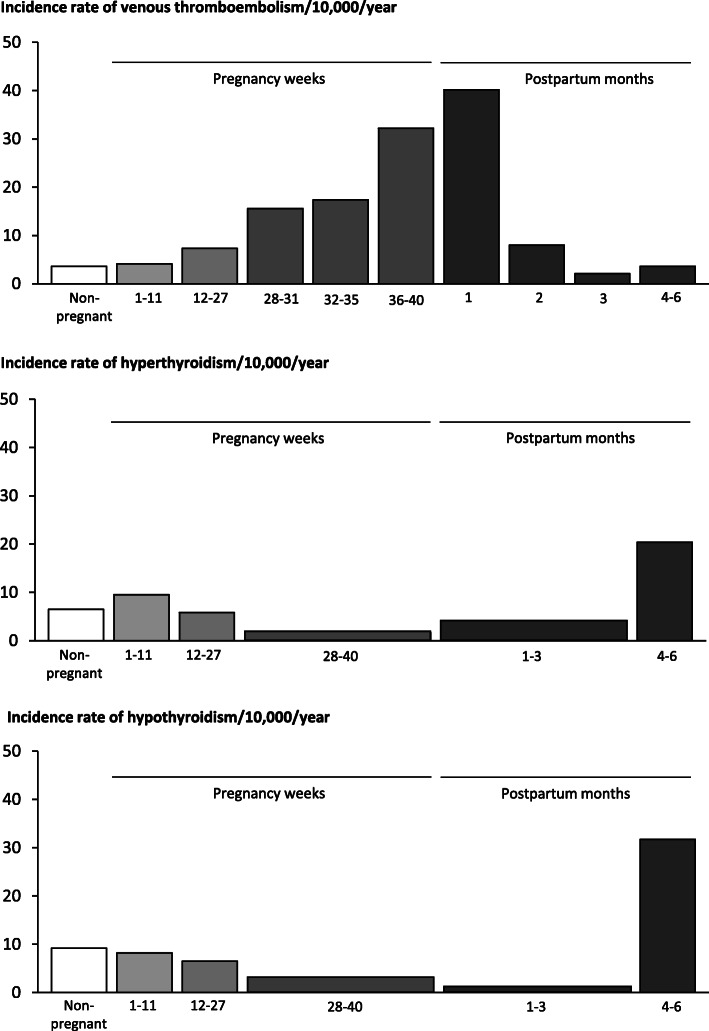
Illustration of the incidence rates of venous thromboembolism, hyperthyroidism, and hypothyroidism in and around pregnancy. The illustration is made using data from Danish nationwide investigations [1, 6, 7]

Due to the physiological changes associated with pregnancy, the risk of VTE in pregnant women is 4–6-fold increased [1]. Besides the changes in the hemostatic system, the distension of the uterus may cause a compression on the veins in pelvis and most pregnancy-related DVTs are localized in the left lower extremity [26]. In addition, personal factors such as obesity, the presence of thrombophilia, age, comorbidities (e.g. inflammatory bowel disease) and complications during pregnancy (e.g. preeclampsia, gestational diabetes, and caesarean section) increase the risk [2, 27]. The incidence has been calculated in some reviews to 1.1–1.2 per 1000 deliveries [2, 3], but with a substantial variation between studies depending on the populations. It is a characteristic that the incidence of DVT in pregnancy varies with gestational age [1, 2]. Thus, the incidence is steadily increasing in pregnancy with increasing gestational age and peaks around birth of the child (Fig. 2, upper part). In the postpartum period, the incidence of DVT is high immediately after birth of the child and declines within the first and second postpartum month reaching non-pregnant levels in the third month (Fig. 2, upper part).

## Thyroid function in pregnancy

Thyroid diseases are common endocrine disorders and part of the chronic disease burden in pregnant women [28]. Thyroid hormones are important developmental factors and play a crucial role during fetal brain development [29]. The fetal thyroid gland is increasingly able to synthesize thyroid hormones from mid-pregnancy, but adequate levels of maternal thyroid hormones is important to ensure supply of thyroid hormones to the fetus throughout the pregnancy [30, 31]. The physiological changes during a normal pregnancy affect the thyroid gland and challenge the evaluation of maternal thyroid function in pregnancy [4]. As in non-pregnant individuals, thyroid function is assessed from the measurement of thyroid stimulating hormone (TSH) and the thyroid hormones thyroxine (T4) and triiodothyronine (T3). The majority of circulating T4 and T3 is protein-bound and predominantly bound to thyroxine binding globulin (TBG). T4 and T3 can be evaluated from the measurement of the total or the free thyroid hormone concentration. As in non-pregnant individuals, overt hyperthyrodism and hypothyroidism are defined by TSH and T3/T4 outside the reference ranges, whereas subclinical disease is characterized by isolated abnormalities in TSH. However, non-pregnant reference ranges cannot be used for the evaluation of maternal thyroid function in pregnancy, and pregnancy specific reference ranges should preferably be established and used [32].

**Fig. 3:**
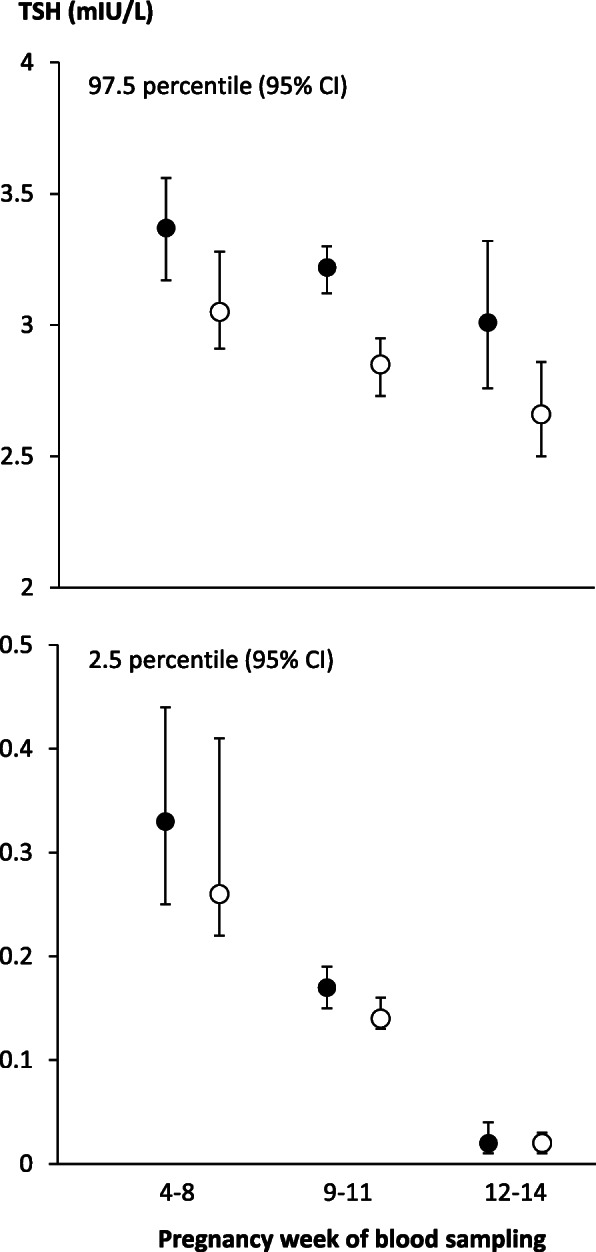
Illustration of the upper (97.5 percentile) and lower (2.5 percentile) reference limit with 95% confidence interval for maternal thyroid stimulating hormone (TSH) in early pregnancy stratified by weeks of pregnancy. Data are from a regional Danish investigation in which maternal TSH was assessed using different biochemical methods (filled circles: Cobas 8000, Roche Diagnostics, open circles: ADVIA Centaur XPT, Siemens Healthineers) [34]

Much emphasis has been on the use of trimester specific reference ranges, but more recent data indicate that maternal TSH is dynamic even within the first trimester of pregnancy [33, 34]. Firstly, the rising estrogen levels in early pregnancy cause an increase in TBG via the effect of estrogen on the liver. This in turn is followed by a concomitant gradual increase in maternal total T4 and total T3 concentrations. This physiological alteration in the total thyroid hormone concentrations has led to the use of free thyroid hormone measurements in many settings. However, automatic immunoassays used in clinical laboratories for the measurement of free T4 and free T3 are indirect methods with no initial separation of free and protein-bound hormone. Thus, the methods are prone to alterations in binding proteins, also in pregnant women, and variation between different assays may be seen [34]. Secondly, hCG shows structural similarities with TSH enabling it to stimulate the thyroid gland to an increased production of thyroid hormones. This mechanism tends to suppress TSH in early pregnancy and this physiological effect is most pronounced in the last part of the first trimester, when the hCG concentration peaks (Fig. 3). A third physiological mechanism is in play in the early pregnancy. The type 3 deiodinase (DIO3) is an enzyme that catalyzes the conversion of T4 to reverse T3 and T3 to T2, thus, inactivating thyroid hormones [35]. This enzyme is expressed in placenta from the early pregnancy and is thought to protect the fetus against excessive transport of thyroid hormones [36, 37]. The activity of the enzyme tends to increase maternal TSH in the early pregnancy (Fig. 3), and maternal TSH in the first trimester may reflect the balance between the DIO3 activity and the hCG effect [4, 33, 34]. Thus, TSH is initially at pre-pregnancy levels followed by a gradual decline with increasing gestational age within the first trimester of pregnancy (Fig. 3) [33].

## Thyroid disease in and after a pregnancy

Thyroid diseases in women of fertile age are predominantly of autoimmune origin [38, 39]. In addition to the physiological alterations in maternal thyroid function, the pregnant state affects the immune system. There is a characteristic immune suppression in pregnancy to allow for the development of the fetus and subsequently an immune rebound after birth of the child. Such alterations may affect the development of autoimmune diseases in and after pregnancy (Fig. 2, lower parts) [6, 7]. Autoimmune thyroid diseases are characterized by the presence of thyroid autoantibodies. Autoimmune hyperthyroidism, also known as Graves’ disease (GD), is associated with TSH-receptor antibodies (TRAb) [39], whereas autoimmune hypothyroidism, also known as Hashimotos’ thyroiditis, is associated with thyroid peroxidase (TPO) and thyroglobulin (Tg) antibodies [38]. Thyroid autoimmunity tends to diminish during pregnancy with a decline in the incidence of autoimmune thyroid disease, while an immune rebound mechanism after birth may trigger onset of disease (Fig. 2, lower parts) [6, 7]. The hyperthyroidism of GD should be distinguished from gestational hyperthyroidism caused by hCG in early pregnancy and from hyperthyroidism as part of postpartum thyroiditis after birth of the child [4]. Gestational hyperthyroidism and hyperthyroidism as part of postpartum thyroiditis are transient disorders and should typically not be treated with antithyroid drugs [32]. The diagnosis may be difficult, however, the presence of TRAb and persistent hyperthyroidism favor a diagnosis of GD [32].

## Thyroid disease and VTE in non-pregnant individuals

Thyroid hormones have many physiological effects and regulate growth, development, and metabolism [29, 30]. It has long been considered whether alterations in the levels of thyroid hormones could disturb the coagulation system. Historically, it has been the view that an increased level of thyroid hormones is associated with a procoagulant condition and vice versa for a low level of thyroid hormones. One of the earliest investigations was published in 1965 by Simone et al. who showed high and low levels of some coagulation factors in 25 hyperthyroid patients and 7 hypothyroid patients, respectively [40]. Several studies have later been performed on this subject, and reviews have drawn the same conclusion [8–10]. In 2012, Stuijver et al. concluded in a systematic review and meta-analysis that hyperthyroidism was associated with a procoagulant, hypofibrinolytic condition with increased levels of factors VIII and IX, fibrinogen, vWF, and PAI-1 [8], and these changes actually resemble to some extent the physiological changes during pregnancy. Prior to this report, a systematic review published in 2007 by Squizzato et al. reported that changes in hypothyroidism was in the opposite direction [9].

In accordance with these findings, hyperthyroidism has been associated with an increased risk of VTE in large observational studies from different countries (Table 1) [41–47]. The studies differed in design and method of exposure assessment, thus, some studies relied on actual measurement of thyroid function parameters in blood samples, whereas others used an indirect measure of exposure from registration of hospital diagnoses. Consequently, it varied between the studies whether information on the subtype of hyperthyroidism was included, e.g. whether it was of autoimmune origin as part of GD. These methodological differences may challenge the direct comparison of individual studies, however, more than half of the studies consistently reported an association between hyperthyroidism and VTE. In support of such an association, some studies also evaluated the association between thyroid hormone levels and outcome of VTE (Table 1) [48–51]. In a population-based cohort study, Lerstad et al. found no association between levels of TSH within the normal range and future development of VTE during 8 years of follow-up, whereas individuals with low and high TSH had a slightly higher risk [48]. Debeij et al. showed in a cohort study and later in a case-control study that the risk of VTE increased with increasing free T4 levels, even within the upper end of the reference interval [49, 50]. The case-control study confirmed that vWF, factors VIII and IX, and fibrinogen increased gradually as free T4 increased, and that the concentration of these proteins, except for fibrinogen, were low at low free T4 levels [49].

**Table 1:** Observational studies on the association between thyroid function and outcomes of venous thromboembolism (deep venous thrombosis and/or pulmonary embolism) in non-pregnant individuals

Author	Year^a^	Country	Design	Population (n)	Exposure assessment	Type of exposure	Association^b^
Hyperthyroidism							
Dekkers et al. [41]	2017	Denmark	Cohort	932,913	Hospital diagnoses	Unspecified	↑
Segna et al. [42]	2016	Schwitzerland	Cohort	561	Biochemical	Subclinical	↓
Zöller et al. [43]	2012	Sweden	Cohort	535,538	Hospital diagnoses	Unspecified	↑
Ramagopalan et al. [44]	2011	United Kingdom	Cohort	4,310,042	Hospital diagnoses	Unspecified	↑
Lin et al. [45]	2010	Taiwan	Case-cohort	53,418	Hospital diagnoses	Unspecified	↑
Danescu et al. [46]	2009	United States	Cohort	908,805,000	Hospital diagnoses	Unspecified	–
Squizzato et al. [47]	2007	Italy	Cross-sectional	150	Biochemical	Overt and subclinical	–
Hypothyroidism							
Segna et al. [42]	2016	Schwitzerland	Cohort	561	Biochemical	Subclinical	–
Zöller et al. [43]	2012	Sweden	Cohort	535,538	Hospital diagnoses	Unspecified	↑
Ramagopalan et al. [44]	2011	United Kingdom	Cohort	4,869,188	Hospital diagnoses	Unspecified	↑
Danescu et al. [46]	2009	United States	Cohort	928,324,000	Hospital diagnoses	Unspecified	↑
Squizzato et al. [47]	2007	Italy	Cross-sectional	150	Biochemical	Overt and subclinical	↑
Thyroid homone levels							
Lerstad et el [48].	2015	Norway	Cohort	11,962	Biochemical	TSH	↑
Debeij et al. [49]	2014	Netherlands	Case-control	5003	Biochemical	TSH and free T4	↑
Debeij et al. [50]	2012	Norway	Cohort	1991	Biochemical	TSH and free T4	↑
van Zaane et al. [51]	2009	Netherlands	Case-control	569	Biochemical	TSH, free T4, total T3	↑

*Abbreviations*: *TSH* Thyroid stimulating hormone, *T4* Thyroxine, *T3* Triiodothyronine

^a^Year of publication

^b^Indicates whether an association between the type of exposure and outcome of venous thromboembolism was observed, see text for details. ↑ higher risk, ↓ lower risk, − no association

In line with these latter findings regarding low levels of thyroid hormones, reports on outcomes of hypothyroidism have predominantly described an increased risk of bleeding in these patients [52], and a quite substantial bleeding risk during treatment with warfarin [53]. Several reports, mainly case-reports, have described an acquired von Willebrand disease (aVWD), i.e. a low level of vWF, in hypothyroid individuals [54]. A study of consecutive hypothyroid patients found that one third of these patients had aVWD, and this may be the main cause of bleeding [55]. The condition appears to be reversible as treatment with Levothyroxine may normalize levels of vWF in a substantial part of the patients [55]. In contrast to this general notion on hypothyroidism and the coagulation system, there are also reports evaluating the risk of VTE in hypothyroid individuals (Table 1) [42–44, 46, 47]. Like the observational findings regarding hyperthyroidism, most of these studies reported an increased risk of VTE associated with hypothyroidism. All studies were observational in design and attempts were made to include potential confounders in multivariate analyses. However, hypothyroidism and VTE share some of the same risk factors, e.g. obesity, which may influence results [56, 57]. On the other hand, smoking which is a risk factor for VTE, seems to protect against autoimmune hypothyroidism [58, 59], illustrating the complexity in the evaluation of various risk factors for VTE. Another consideration is on the autoimmune origin of thyroid diseases, and one may speculate on the role of abnormal thyroid function as opposed to thyroid autoimmunity per se*.* Autoimmune diseases are a heterogenous group of disorders with multifactorial etiology. Many autoimmune diseases show female predominance and many of the diseases affect women of fertile age [60]. Studies looking at a spectrum of autoimmune diseases found an increased risk of VTE in all the autoimmune disorders that they investigated [43], suggesting that autoimmune mechanisms may play a role also in the association between thyroid disease and VTE. Adding to this, autoimmune thyroid diseases overlap with other autoimmune diseases including endocrine disorders (type 1 diabetes, Addison’s disease) and rheumatological disorders (systemic lupus erythematosus, rheumatoid arthritis) [61]. One may speculate on underlying shared genetic risk factors for thrombosis and on underlying pathophysiological alterations in the immune system that cause a prothrombotic condition. A key mechanism in many autoimmune disorders is the presence of autoantibodies, and the autoantibodies characteristic of autoimmune thyroid disease, e.g. TPO-antibodies, are more commonly found in other autoimmune diseases [61]. Antiphospholipid antibodies and platelet antibodies are some of the main autoantibodies described in relation to autoimmune disorders, which may present with thrombosis and/or bleeding symptoms (e.g. antiphospholipid syndrome and idiopathic (or immune) thrombocytopenic purpura) [62, 63]. A possible overlap between these autoimmune disorders and autoantibodies with autoimmune thyroid diseases is an interesting pathophysiological thought and a relevant topic for future research.

In summary, hyperthyroidism in non-pregnant individuals is a procoagulant condition associated with an increased risk of VTE. Hypothyroidism may be associated with a lower activity in the coagulation system and therefore a tendency to increased risk of bleeding but is also associated with an increased risk of VTE.

## Thyroid disease and VTE in pregnant individuals

Moving from non-pregnant to pregnant individuals, it is relevant to consider if similar associations between thyroid disease and VTE exist in pregnancy. As described, there are numerous physiological alterations in and around pregnancy that affect maternal thyroid function and the function of the coagulation system. No original studies have yet investigated the interrelationship between pregnancy, VTE, and thyroid disease, but the incidence of the diseases in and after a pregnancy have been considered separately [1, 6, 7]. In both disorders, pregnancy and the postpartum period seem to have a strong influence on the onset of disease, but the timing of the incidence peak varies (Fig. 2). The incidence of VTE is increasing in pregnancy and peaks just around birth of the child and in the early postpartum weeks. On the other hand, the incidence of hyperthyroidism and hypothyroidism declines with the length of pregnancy and peaks 4–6 months postpartum (Fig. 2). These incidence data emerged from large population-based register-studies using different cohorts, and thyroid disease and VTE were not evaluated in the same individuals. However, each study included enough women to provide estimates stratified by short time periods within and after a pregnancy, and the figures (Fig. 2) provide clues on the pattern of incidence variation for each disorder in relation to the pregnancy period. It should be noted that the identification of disease using such design, relied on hospital diagnoses and redeemed prescriptions of drugs. Thus, the onset of disease is a proxy defined from these data, and actual onset may have preceded the date of diagnosing or initiation of treatment, especially for thyroid diseases, which typically present less acute [6, 7].

We can only speculate on possible mechanisms behind an interplay between thyroid disease and VTE in pregnancy. Obviously, the incidence of the diseases in and around pregnancy are not changing in parallel (Fig. 2), but from the findings in non-pregnant individuals, we speculate whether abnormalities in maternal thyroid function in pregnancy could exacerbate the physiologically altered coagulation function in pregnant women. Thyroid hormones regulate the transcription of proteins including the coagulation proteins [64]. Evidence from non-pregnant individuals indicate that thyroid hormones may cause elevated coagulation factors in hyperthyroid patients [8]. Furthermore, thyroid hormone alterations may affect the synthesis of sex hormones, and elevated concentrations of estradiol and the sex-hormone-binding-globulin may be seen in hyperthyroidism [65]. This alteration may further enhance the effect of estradiol on the coagulation system.

In summary, an association between thyroid disease and VTE in pregnant women seems biologically plausible, particularly regarding hyperthyroidism. As in non-pregnant individuals, autoimmune mechanisms could also play a role and shared environmental risk factors between thyroid disease and VTE should be considered as part of the hypothesis.

## Clinical implications

Our aim with the present review was to describe the hypothesis of an association between thyroid disease and VTE in pregnancy, specifically, and to enhance future scientific work on this subject. If an association is established, a perspective of prevention and clinical implications will follow. Clinical uncertainties exist regarding both disorders. For thyroid disease in pregnancy, the benefits and risks of routine testing of thyroid function are not clarified, and a risk-based screening is currently recommended [32]. Considering treatment of thyroid disease in pregnancy, overt hyper- and hypothyroidism should be treated to prevent maternal and fetal complications, but for subclinical hyperthyroidism there is no recommendation of treatment and for subclinical hypothyroidism, the indication for treatment depends on the combined assessment of TSH levels and the presence of TPO-antibodies [32]. From studies in non-pregnant individuals it seems uncertain whether associations with VTE primarily occur in overt or also in subclinical thyroid disease [42, 48], and the distinction between subtypes of thyroid function abnormalities is an important focus of future research in pregnant and in non-pregnant individuals. For the management of VTE in pregnant women, the main clinical decision is on the indication for thromboprophylaxis [66]. This decision is based upon an assessment of individual risk factors including the presence of thrombophilia and other medical conditions. If an association between thyroid disease and VTE in pregnancy is established, a future clinical perspective is whether women with thyroid disease are considered at high risk for thrombosis and would benefit from thromboprophylaxis in the pregnancy, especially as an additional risk factor in women with other risk factors.

## Conclusions

Substantial evidence suggests an association between abnormal thyroid function and abnormalities in the coagulation system in non-pregnant individuals, and an increased risk of VTE has been proposed in hyperthyroid and hypothyroid patients. Thyroid disease and VTE are a matter of concern in pregnant women, and the physiological changes in pregnant women affect the incidence of the disorders. To our knowledge, no studies have yet investigated the interrelationship between pregnancy, VTE, and thyroid disease. Future scientific work should enhance our understanding of a potential association between thyroid disease and VTE in pregnant women specifically. Expanding future work to include the biochemical assessment of thyroid hormone parameters, autoantibodies, coagulation proteins, and sex-hormones could shed light on the relationship, and the potential role of autoimmune mechanisms and environmental factors should preferably also be considered.

## Data Availability

Data sharing is not applicable to this article as no datasets were generated or analysed during the current study.

## Abbreviations

APTT: Activated partiel thromboplastin time
aVWD: Acquired von Willebrand disease
DIO3: Type 3 deiodinase
DVT: Deep venous thrombosis
GD: Graves’ disease
hCG: Human chorionic gonadotropin
PCa: Activated protein C
PAI-1: Plasminogen activator inhibitor-1
PAI-2: Plasminogen activator inhibitor-2
PS: Protein S
PT: Prothrombin time
TBG: Thyroxine binding globulin
TF: Tissue factor
Tg: Thyroglobulin
t-PA: Tissue-type plasminogen activator
TPO: Thyroid peroxidase
TRAb: TSH-receptor antibodies
TSH: Thyroid stimulating hormone
T3: Triiodothyronine
T4: Thyroxine
u-PA: Urokinase-type plasminogen activator
VTE: Venous thromboembolism
vWF: Von Willebrand factor
